# Effectiveness of Oral Hygiene Instructions Given in Computer-Assisted Format versus a Self-Care Instructor

**DOI:** 10.3390/dj6010002

**Published:** 2018-01-10

**Authors:** Kristin A. Williams, Sara Mithani, Ghazal Sadeghi, Leena Palomo

**Affiliations:** 1 Department of Community Dentistry, Case Western Reserve University, Cleveland, OH 44106, USA; kaw14@case.edu; 2 College of Nursing, University of Illinois Chicago, Peoria, IL 61605, USA; smitha3@uic.edu; 3 Department of Periodontics, Case Western Reserve University, University, Cleveland, OH 44106, USA; gxs365@case.edu

**Keywords:** oral health, self-care, computer assisted instruction

## Abstract

Background: This randomized, single-blinded, controlled study compares the effectiveness of two modes of delivering the same set of oral hygiene instructions: those loaded on a computer-assisted teaching format versus those given personally by a self-care instructor. Methods: 60 participants with mild to moderate periodontitis were randomized to either the computer-teaching format or the self-care instructor. Plaque score and bleeding indices were assessed at baseline and at 4 week re-evaluation to compare the instructional modalities. Results: At baseline, there was no difference in the parameters between the two groups. At re-evaluation, all parameters were significantly improved compared to baseline; however, there was no difference between any parameters in the computer group versus the instructor. Plaque score was significantly different between younger and older participants, such that participants under 50 years old had lower plaque scores when they received instructions using the computer format (72.5 ± 12.6 vs. 87.2 ± 10.4; *p* < 0.001). Additionally, in those under-50 year olds, plaque scores were significantly better in the group using the computer format (87.2 ± 10.4 vs. 78.3 ± 15.6; *p* = 0.03). Conclusion: Computer teaching formats and self-care instructors are both effective means of communicating oral hygiene instructions. Computer-assisted instruction format effectiveness may amplify as the population ages. Applications of computer formats teaching oral hygiene instructions and cost effectiveness comparison studies warrant significant future investigation.

## 1. Introduction

Host response to accumulated plaque bacterial biofilm causes periodontal disease [1,2]. Effective self-performed regular oral hygiene has been identified as a key step in disease prevention [3]. Prevention strategies are valuable in light of reports that the periodontitis prevalence in US adults remains at 46%; and poor oral hygiene and compliance have been identified as risk factors in periodontitis pathogenesis [4,5]. Effectiveness of oral hygiene instructions, then, plays a key role in prevention, beyond professional treatment [6].

Over the years, oral hygiene instructional practices have been investigated. However, most studies focus on cognition and psychology related to motivation, rather than instructional methodology [7,8]. There is a paucity of investigation around best practices in oral hygiene instruction. A more recent study focused on instructional methodology and compared written and oral instruction [9]. Such written instructional material, with illustrations, has a long history of being used, dating back to a pamphlet published in 1969 by O’Leary and Nabors [10]. Despite the fact that, in the previous decades, the evolution of multimedia software applications has far outpaced printed pamphlets, computer-assisted teaching formats have not been rigorously evaluated. Furthermore, even though video instructional formats have been shown to be effective in teaching mentally challenged subjects oral hygiene skills, even when using manual tooth brushes, multimedia software implementation in a computerized teaching format has not been thoroughly studied [11,12]. In fact, a recent systematic review seeking to compare computer-aided learning in oral health underscored the lack of randomized controlled trials, and called for more investigation [13]. In light of the expanding use of tablets, phones and laptops, the effectiveness of computerized teaching formats on learning oral hygiene warrants study. Such computer-assisted instruction formats have demonstrated effectiveness in other areas of self-care. For example, randomized clinical trials to teach self-care behaviors to patients with Type 2 diabetes and to teach self-examination for melanoma have been shown to be efficacious [14,15]. 

This study compares the effectiveness of a computerized teaching format versus a self-care instructor format for oral hygiene instruction. 

## 2. Methods

60 participants were randomized into two groups for this Investigational Review Board of Case Western Reserve University-approved study. One group received oral hygiene instructions in a computer-teaching format, the other from a self-care instructor. 

### 2.1. Participants

Participant demographics are given in Table 1. Participants were recruited from a institutional dental clinic waiting area. Inclusion criteria included 1. Mild to moderate periodontitis, with non-surgical periodontal therapy planned for treatment; and 2. All pockets <6 mm. Exclusion criteria included: 1. Smokers; 2. Uncontrolled diabetics (HbA1C(meaure of glycosylated hemoglobin) >7); 3. Use of blood thinners, including over-the-counter aspirin; 4. Physical incapacity to use a manual toothbrush or floss (such as, but not limited to, paralysis); 5. Bleeding/clotting disorders; 6. Deafness; and 7. Blindness.

### 2.2. Test Conditions

Baseline variables were recorded from the dental charts, which were obtained by a blinded clinician who was calibrated for inter- and intra-examiner reliability (*p* < 0.05) prior to the start of non-surgical therapy visit. The clinician was a trained, experienced and board-certified periodontist. At the completion of the non-surgical therapy visit, participants in the computer-teaching format group were given a tablet personal computer (PC) containing a slide presentation designed to teach oral hygiene instructions, brushing and flossing. 

The computer format group received an 8 min audio-visual Powerpoint presentation containing 12 slides. 6 slides teach and demonstrate the modified Bass technique for brushing, and 6 slides teach and demonstrate interdental cleaning, including flossing technique and use of interproximal brushes. Participants were left alone in the cubicle to go through the presentation as many times as they desired; the slides could be reversed and reviewed at the discretion of the participant. When the participant determined completion, (s) he was able to call for dismissal. The second, control group, met with a self-care instructor, who delivered the same instructions, with the same script, and visual aids matching those on the software, but during a personal visit lasting 8 min rather than with the computer-assisted format using a tablet. Participants were free to ask for repetition and/or demonstration of any information. However, questions beyond the scripted information were not answered until after participant outcome assessment the conclusion of the study.

Since oral hygiene instructions typically follow non-surgical therapy appointments, this protocol was adhered to for both groups. 

All participants were asked to practice oral hygiene as taught by the respective instructional format, over the next 4 weeks, and then present for re-evaluation. At re-evaluation, the same blinded calibrated clinician measured the periodontal parameters. 

### 2.3. Variables

Three periodontal parameters, accepted by the American Board of Periodontology, the American Academy of Periodontology a recognized specialty of the American Dental Association were recorded at initial visit and again at re-evaluation for outcome assessment for every erupted tooth. The first of these outcome variables is Plaque Score (PS); a modified O’Leary Index is used to define the number of surfaces free of plaque as a percentage of surfaces in the mouth [16], such that there are 6 surfaces per tooth. Thee bleeding index (BI) of Silness and Loe [17] was used such that a score of 0 indicates no bleeding on probing, 1 indicates a single bleeding point, 2 indicates several bleeding points or thin line, 3 indicates interdental space filled with blood. While this index measures the amount of bleeding, there is some subjectivity in the BI. To counter this subjectivity, dichotomous bleeding on probing percentage (BOP) of sites in the mouth is also measured [18]. Demographic post hoc analysis will also be conducted. Post hoc analysis, by definition, is analysis that was not specified before seeing data. Ad hoc analysis, by definition, is analysis of the data for the purpose of answering the aim of the study. Demographic differences not specified before seeing data warranted further evaluation after the ad hoc analysis is completed. 

### 2.4. Statistical Analysis

Kolmogorov-Smirnov test was used to test for normal distribution. Analysis of variance (ANOVA)as used to evaluate baseline difference (in parametric data); Kruskal-Wallis tests (for non-normally distribution) and Pearson Correlation coefficient were used to evaluate strength of correlation.This is such that *p* < 0.05 is considered significant. SPSS Software (IBM Corporation, Armonk, NY, USA) was used for analysis. 

## 3. Results

Participant demographics are shown in Table 1. Groups did not differ at baseline with respect to any variables (Table 2). At the 4-week re-evaluation visit, two patients did not return. Both of these patients were from the control group; one was a 28-year-old man and one was a 68-year-old woman. At re-evaluation, all parameters—PS, BI and BOP %—were significantly improved in both groups (Table 3). However, there was no significant difference in any parameters between the groups that received computer versus instructor format (Table 4).

Post hoc analysis by age shows significant differences in PS between older and younger participants trained on the computer (72.5 ± 12.6 vs. 87.2 ± 10.4; *p* < 0.001). When older participants from the computer group were compared to older patients from the instructor group, there was no significant difference; however, when younger participants from the computer group were compared to the younger self-care instructor group, there was significant difference. (87.2 ± 10.4 vs. 78.3 ± 15.6; *p* = 0.03) No differences were found based on sex (Table 5). There was no significant age related correlation (r = 0.07, *p* = 0.7). Outcomes are such that younger participants were not significantly different with respect to instructional methodology, however at 4 week re-evaluation; the older participants had lower plaque score when they received instructions from the self-care instructor. 

## 4. Discussion

This study compares two methods of giving oral hygiene instructions to patients after non-surgical therapy: computer-assisted instruction loaded on a tablet versus personally by a self-care instructor. This study finds no difference between any parameters in the computer group versus the instructor. Plaque score is significantly different between younger and older participants, such that participants under 50 years old had lower plaque scores when they received instructions using the computer format (72.5 ± 12.6 vs. 87.2 ± 10.4; *p* < 0.001). Additionally, in those under 50 years old, plaque scores were significantly better in the group using the computer format (87.2 ± 10.4 vs. 78.3 ± 15.6; *p* = 0.03). 

At baseline, the plaque and inflammation measurements in the two groups were not significantly different, allowing for the assumption that the groups are comparable. It is no surprise that our results show that following professional non-surgical therapy, periodontal parameters improve in both groups, regardless of modality for oral hygiene instructions. At re-evaluation 4 weeks after professional therapy, the results in all participants confirm the purpose of professional treatment and oral hygiene instructions is being accomplished [19]. Periodontal parameters are improved versus baseline in both groups. Comparison of the mean outcome variables between groups, like the baseline, is not significantly different. Outcomes in the oral hygiene instructions given via computer format are as effective as those given by a self-care instructor. 

Effectiveness of the computer format implies that an additional professional may not be necessary for teaching self-care. This supports the assertion that using computerized, network connectivity-based oral hygiene instruction may be useful in improving care. Such a format has been noted for its ability to reach patients who have difficulty getting to the dental office, as well as underserved populations [20]. The current findings warrant research into the cost effectiveness of using computer format instructions for oral hygiene and its usefulness in reaching populations who have difficulty accessing clinics, such as the elderly, the homebound, and rural populations. Furthermore, a recent workshop of the European Federation of Periodontology emphasized that there is benefit to reinforcing oral hygiene instructions [21]. A computer format is more easily transferred to the patient, via network connectivity, for such reinforcement. Our study supports exploration of this method. 

Participants under age 50 had significantly better PS in the computer group versus the instructor group. This suggests that the computer learning format usefulness will increase as the population continues to age. 

It is of note that when comparing PS in participants over 50, there was no significant difference between instructional formats. Additionally, there was not a significant correlation between PS and age. This suggests that age, by year, is not a determinant, but rather a generational style or aptitude, which may not coincide directly with chronologic age is responsible. 

One reported strength in computer-assisted learning over other methods is that it allows for self-directed learning protocols [22]. This self-directed approach is accommodated in the computer format group. The software allows for forwarding and reversing slides for the purpose of real-time clarification and understanding, rather than having to request another person to do so. One limitation of this study is that random errors may interfere with learning. For example, the visual distraction of flash clinical photography in an adjacent operatory may trigger a participant to repeat a slide or sound interference from a conversation in an adjoining cubicle may trigger participant request for more information from the instructor. The current study design treats random error with the standard assumption that its effects cannot be tracked. 

Similarly, the current findings do not support the assertion that there is a sex-based difference in understanding computer format versus personally delivered oral hygiene instructions. 

Another strength of the computer format that warrants more investigation is its usefulness in elderly patients with comprehension deficits. It is of note that comprehension deficits reported in older patients have a domain-dependent relationship. For instance, males are associated with non-comprehension of diet instructions, and social isolation is associated with non-comprehension of exercise instructions [23,24]. The current findings do not prove such deficits, as periodontal parameters in older participants improved regardless of instructional modality. The variation in outcomes is significantly different, based on the method of teaching, only in the under-50 cohort. Investigations in internal medicine assert that in elderly patients, lack of comprehension in hospital discharge instructions remains a poorly understood challenge. Health literacy, cognition and self-efficacy have been identified as important predictors of executing self-care instructions when it comes to being discharged from a hospital [25]. It is reported that comprehension deficits, reported among older patients can reportedly be overcome through follow-up [26]. Our findings strengthen the need to investigate whether a computer teaching format may be useful in such patients, where computer learning can be retrieved repeatedly on a self-directed basis. 

PS stands out compared to bleeding measurements such as BOP and BI because it is directly related to the effect of oral hygiene on plaque accumulation. Bleeding, on the other hand, is multifactorial and may be affected by other factors [27]. Neither inflammation parameter—BI or BOP—differed between younger and older participants between the two groups. Classic investigations have shown that oral hygiene has a limited effect on inflammation in deeper pockets, whereas bleeding is well known to be related to other factors such as probing force, probing frequency etc. [28,29,30]. In spite of emphasis placed on the need for objective signs of inflammatory changes in the periodontium, bleeding never-the-less remains the most objective clinical sign available, due in part to histologic studies that relate the dilated vessels to bleeding on slight manipulation of the probe [31]. Our results support the assertion that this may not always be the case. Although the primary etiology of periodontitis is host response to plaque, that host response inflammatory response may also vary based on host centered factors in the immune response [32]. This may account for why the differences between the groups are consistent for indexes of inflammation. 

Limitations of this study include random error related to sight and sound related distractions which may impact learning. With random effects controlled, comparing the number of repetitions in both formats may be useful in understanding the differences. In this study the defects were not matched in the two groups. Comparing the two formats in a defect-matched sample would account for baseline differences not accounted for by this study. This study employed clinical outcomes which are subject to bias, even though these are the paramount indexes of the specialty of Periodontology. Histologic studies to evaluate inflammatory cell burden would more objectively identify inflammation. 

## 5. Conclusions

Computer-assisted instructional formats and self-care instructors are both effective means of teaching oral hygiene instructions. Computer-assisted learning holds promise in prevention of periodontitis. This role may be amplified as the population ages. Applications of computer-assisted instructional formats teaching oral hygiene instructions warrant and cost effectiveness comparison studies significant future investigation. 

## Figures and Tables

**Table 1:** Participant demographics.

Factor	Total
**Race/Ethnicity**	60
White	25
Black	28
Hispanic	2
Other	5
**Age**	
21–30	12
31–40	9
41–50	9
51–60	11
61–70	13
71–80	6
**Sex**	
Male	22
Female	38

**Table 2:** Mean and standard deviation periodontal parameters at baseline.

	Computer *n*: 30	Instructor *n*: 30	*p*
PS	68 ± 10.7	65.8 ± 7.1	0.3
BI	0.28 ± 0.1	0.26 ± 0.1	0.4
BOP % of sites in the mouth	42 ± 15.3	37.8 ± 15.2	0.3

**Table 3:** Mean and standard deviation periodontal parameters at re-evaluation.

	Baseline	Re-Eval	*p*	Baseline	Re-Eval	*p*
Instructional Format	Computer *n*: 30	Computer *n*: 30		Instructor *n*: 30	Instructor *n*: 28	
PS	68 ± 10.7	79.8 ± 11.4	0.0005	65.8 ± 7.1	76.5 ± 11.9	0.0001
BI	0.28 ± 0.1	0.23 ± 0.09	0.04	0.26 ± 0.1	0.17 ± 0.1	0.002
BOP % of sites in the mouth	42 ± 15.3	32.2 ± 20.9	0.04	37.8 ± 15.2	30.6 ± 10.7	0.04

**Table 4:** Mean and standard deviation computer learning versus self-care instructor format at re-evaluation.

Instructional Format	Computer *n*: 30	Instructor *n*: 28	*p*
PS	79.8 ± 11.4	75.6 ± 11.3	0.16
BI	0.23 ± 0.09	0.17 ± 0.1	0.06
BOP % of sites in the mouth	32.2 ± 20.9	30.6 ± 10.7	0.70

**Table 5:** Ad hoc analysis of mean and standard deviation periodontal parameters comparing male versus female and younger versus older participants within in computer and instructor groups.

Parameter	Computer *n*: 30		*p*	Instructor *n*: 28		*p*
	Male *n*: 10	Female *n*: 20		Male *n*: 11	Female *n*: 17	
PS	75 ± 12.3	82.2 ± 11	0.1	77.3 ± 13.6	74.3 ± 9.8	0.92
BI	0.18 ± 0.1	0.24 ± 0.1	0.09	0.15 ± 0.08	0.18 ± 0.13	0.5
BOP % of sites in the mouth	29.0 ± 14.5	33.8 ± 38.6	0.07	31.1 ± 7.5	30.3 ± 12.7	0.85
	Older *n*: 15	Younger *n*: 15		Older *n*: 14	Younger *n*: 14	
PS	72.5 ± 12.6	87.2 ± 10.4	*p* < 0.001	73 ± 7.5	78.3 ± 15.6	0.24
BI	0.26 ± 0.10	0.19 ± 0.13	0.07	0.20 ± 0.1	0.16 ± 0.10	0.5
BOP % of sites in the mouth	37.7 ± 17.5	26.2 ± 24.3	0.07	35.5 ± 9.2	26.9 ± 12.4	0.05
